# Vestibular hair cell redundancy and the critical requirement of type I cells for balance maintenance

**DOI:** 10.1007/s00018-026-06348-1

**Published:** 2026-07-17

**Authors:** Yikang Huang, Wenli Ni, Qin Zhou, Mingchuan Feng, Tao Jiang, Shunji He, Yan Chen, Wenyan Li

**Affiliations:** 1 ENT institute and Department of Otorhinolaryngology, Eye & ENT Hospital, State Key Laboratory of Medical Neurobiology and MOE Frontiers Center for Brain Science, Fudan University, Shanghai, 200031 PR China; 2 NHC Key Laboratory of Hearing Medicine, (Fudan University), Shanghai, 200031 PR China

**Keywords:** Vestibular function, Balance, Hair cells, Cell subtype, Mouse model

## Abstract

Vestibular hair cells (HCs) are sensory mechanotransducers essential for balance and spatial orientation, yet the relative contributions of HC number and subtype to vestibular function remain unresolved. Here, we developed a dose-dependent injury model in adult *Pou4f3*^DTR^ mice by administering diphtheria toxin (DT), which induced selective HC ablation across vestibular organs. Both type I and type II HCs were lost following DT treatment. Type II HCs exhibited higher susceptibility to DT at lower doses, although this difference diminished as DT dose increased and overall HC survival declined. Functional analyses revealed that mice retaining ~ 30% of vestibular HCs—with preserved subtype proportions and neural innervation—exhibited normal balance, while ~ 50% residual HCs were sufficient to maintain intact vestibulo-ocular reflexes. To elucidate subtype-specific roles, we employed two type I HC–deficient models: 3,3′-iminodipropionitrile-induced injury and *Gfi1*^Cre^/*Atoh1*-OE mice. Comparative analyses revealed that type I HCs were essential for vestibular function. Together, these findings establish a reliable injury model, demonstrate functional redundancy within vestibular HCs, and underscore the critical role of type I HCs. These insights suggest that efforts to restore vestibular function through HC regeneration should prioritize generating functional type I HCs, not merely increasing the total HC count.

**Graphical Abstract:**

**Figure:**
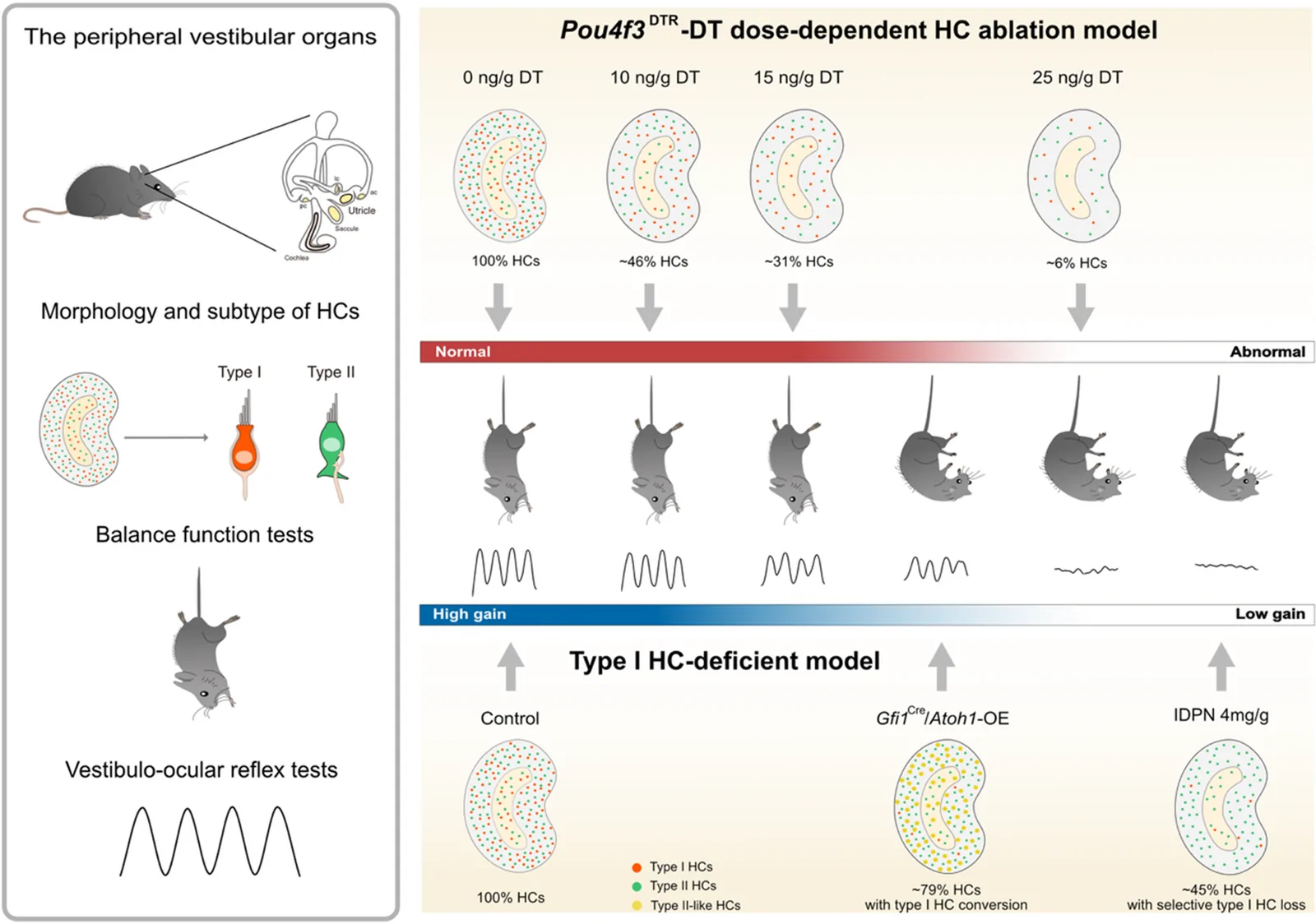

**Supplementary Information:**

The online version contains supplementary material available at https://doi.org/10.1007/s00018-026-06348-1.

## Significance Statement

Established a dose-dependent, HC-specific vestibular injury model enabling precise functional assessment.

Revealed functional redundancy of vestibular HCs to balance maintenance.

Identified type I HCs as essential for vestibular function, guiding targeted regenerative strategies.

**Supplementary Information:**

The online version contains supplementary material available at https://doi.org/10.1007/s00018-026-06348-1.

## In brief

Using novel vestibular injury models, the authors identify functional redundancy in the system—balance requires only 30% of hair cells (HCs), and vestibulo-ocular reflexes require only 50%. Furthermore, they demonstrate that vestibular function has a critical dependence on the preservation of type I HCs, not merely on the total HC number.

**Supplementary Information:**

The online version contains supplementary material available at https://doi.org/10.1007/s00018-026-06348-1.

## Introduction

Body balance and spatial orientation rely on the integration of vestibular, visual, and proprioceptive inputs. Mammalian vestibular organs are located in the inner ear. Vestibular hair cells (HCs), which act as mechanosensory transducers, are located on top of supporting cells in the vestibular sensory epithelium and are responsible for detecting and transducing mechanical stimuli into electrical signals that are relayed to the central nervous system. Utricular and saccular HCs can detect linear acceleration and gravity, while crista HCs sense angular acceleration. Each vestibular organ consists of a central and a peripheral zone; in the otolith organs (utricles and saccules), these two zones are referred to as the striola and extrastriola [1–3]. Vestibular HCs are classified into type I and type II based on their morphology and physiology [4, 5]. Type I HCs are flask-shaped and are innervated by nerve terminals called calyxes, while type II HCs possess basolateral cytoplasmic processes and are innervated by smaller bouton-like terminals [4, 6, 7]. Our previous study demonstrated that constitutive *Atoh1* expression in vestibular HCs disrupts type I HC differentiation, producing more type II-like HCs [8]. However, the specific contributions of individual HC subtypes to vestibular function remain poorly understood.

Vestibular HCs are susceptible to damage from various factors such as aging, ototoxic drugs, and infections [9–11]. Such damage can lead to balance disorders, including vertigo and dizziness, which severely affect quality of life [10]. In adult mammals, the spontaneous regenerative capacity of vestibular HCs is insufficient to restore balance function after severe HC loss [12–14]. Previous studies have attempted to promote vestibular HC regeneration by upregulating *Atoh1*, inhibiting Notch or combining glycogen synthase kinase 3β and histone deacetylase inhibitors to induce supporting cell transdifferentiation into HCs [15–18]. However, despite the significant increase in HC numbers, functional recovery—particularly balance—remained minimal. This raises a critical question: how many, and which types, of HCs are necessary to maintain vestibular function? A specific and dose-dependent HC injury model may provide the answer.

Aminoglycoside antibiotics, a class of ototoxic drugs, have been widely used to kill cochlear and vestibular HCs, but achieving in vivo vestibular HC damage typically requires high doses, which can cause systemic toxicity [19–21]. In comparison, 3,3′-iminodiproprionitrile (IDPN), another vestibulotoxic drug, is more suitable for establishing an in vivo vestibular injury model [14, 16, 22–24], but its damage to neural terminals and supporting cells at high doses may lead to an overestimation of the effect of HC loss on vestibular function [14, 25–28]. To overcome these problems, *Pou4f3*^DTR^ mice were generated, in which the human diphtheria toxin receptor (huDTR) is expressed under the *Pou4f3* promoter. Injection of diphtheria toxin (DT) selectively ablates vestibular HCs without apparent systemic toxicity or supporting cell loss [29]. While previous studies typically used high DT doses (≥ 25 ng/g, adult mice) to eliminate over 90% of utricular HCs [12, 29–31], the dose-dependent effects of DT on vestibular HCs remain unclear.

In this study, we established a dose-dependent vestibular HC injury model in adult *Pou4f3*^DTR^ mice by administering varying doses of DT, achieving selective HC ablation across vestibular organs. We then investigated the relationship between the HC number and vestibular function through a series of tests, including balance function tests and vestibulo-ocular reflex tests. Surprisingly, mice retaining approximately 30% of vestibular HCs—with normal subtype proportions and preserved innervation—performed normally in balance-related behavioral assays (open-field tests, anti-gravity reflex tests, and swimming tests). Mice with approximately 50% residual HCs maintained normal vestibulo-ocular reflexes. These findings validate the use of this dose-dependent, HC-specific injury model and highlight the functional redundancy of vestibular HCs. Furthermore, by comparing HC subtype composition and vestibular function across the *Pou4f3*^DTR^–DT model, the IDPN model, and the *Gfi1*^Cre^/*Atoh1*-OE model, we found that the preservation of type I HCs contributes more to balance and vestibulo-ocular reflex than type II HCs. Together, these results suggest that functional recovery depends more on HC quality, especially subtype identity, than on HC quantity alone.

## Results

### DT induced dose-dependent HC death in the utricles of adult mice

**Fig. 1:**
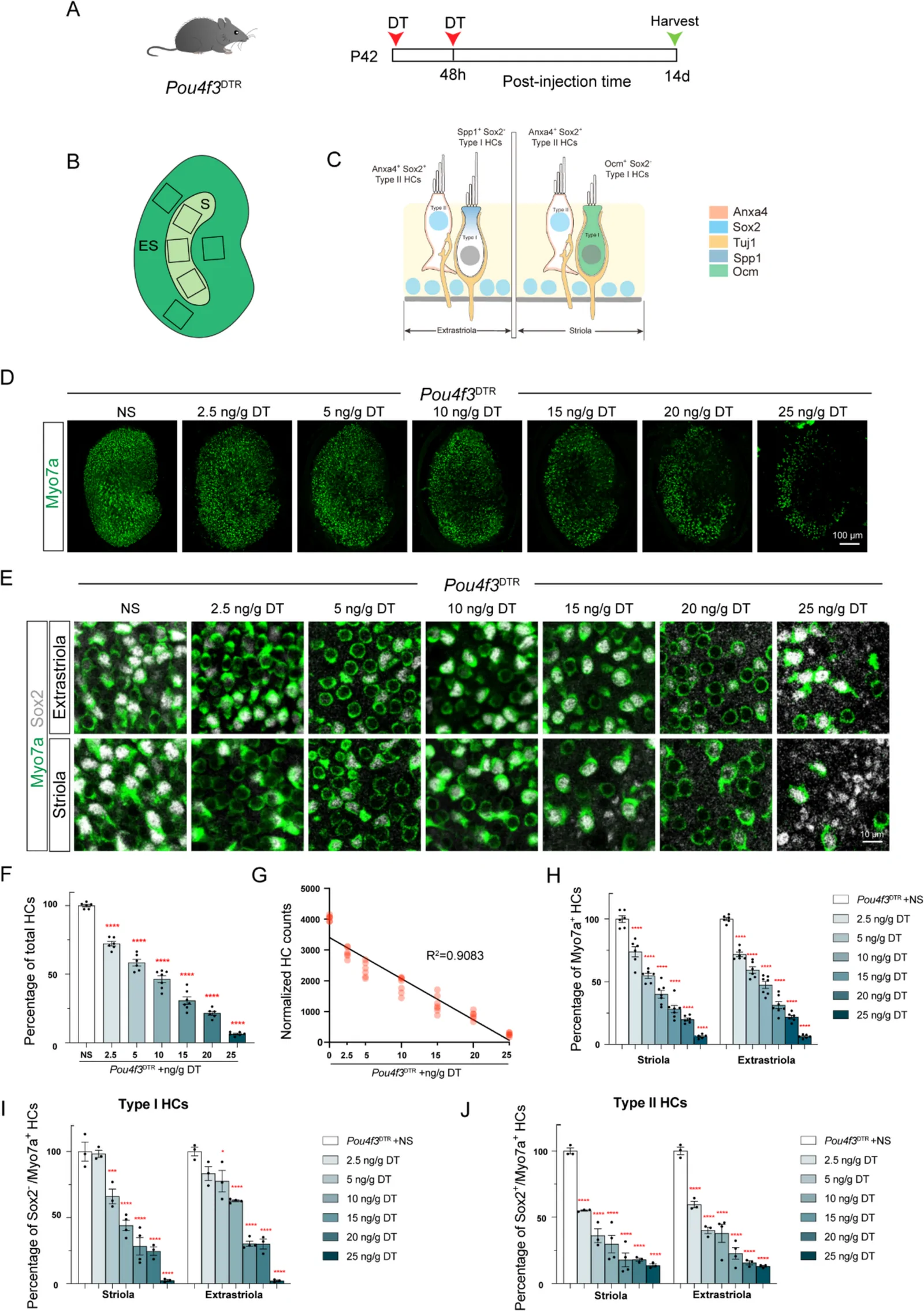
DT injection induced dose-dependent injury of utricular HCs. (**A**) Schematic illustration of the experiment. *Pou4f3*^DTR^ mice received NS or DT at P42, and utricles were harvested 2 weeks later (**B**) Schematic of the utricle and its different zones, including the striola (S) and the extrastriola (ES). Squares denote sampling regions for quantitative statistics (**C**) Schematic depiction of the morphological characteristics and antigen specificities of type I and type II HCs (**D**) Low-magnification images showing a decrease in Myo7a^+^ HC density with increasing doses of DT (**E**) Representative images demonstrating a reduction in Sox2^−^/Myo7a^+^ and Sox2^+^/Myo7a^+^ HCs in both the striola and extrastriola following increasing doses of DT (**F**) Total HC counts expressed as percentages of the control group (*n* = 6 or 7) (**G**) Correlation between the number of residual utricular HCs and the doses of DT (simple linear regression, *p* < 0.0001) (H-J) Myo7a^+^ HC counts (**H**) (*n* = 6 or 7), Sox2^–^/Myo7a^+^ type I HC counts (**I**) (*n* = 3 or 4) and Sox2^+^/Myo7a^+^ type II HC counts (**J**) (*n* = 3 or 4) in the striola and the extrastriola expressed as percentages of the control group Scale bars: 100 μm in D, 10 μm in E. Red asterisks indicate statistical significance compared with the NS group. One-way ANOVA was performed for F, H-J. * *p* < 0.05, ** *p* < 0.01, *** *p* < 0.001, **** *p* < 0.0001

To establish a dose-dependent HC-specific damage protocol, we injected varying doses of DT into transgenic *Pou4f3*^DTR^ mice, in which huDTR expression is driven by the *Pou4f3* promoter [29]. *Pou4f3* is exclusively expressed in HCs of the mouse inner ear from the embryonic to the adult stage [32–35]. In the previous study [29], DT injection in adult *Pou4f3*^DTR^ mice led to about 94% HC loss and severe vestibular dysfunction. However, whether DT-induced HC death is dose-dependent remained unclear. To address this, we injected 6-week-old mice with DT at 2.5, 5, 10, 15, 20, or 25 ng/g, and analyzed HC survival 14 days later (Fig. 1A). Mice receiving normal saline (NS) were used as controls. HC number and distribution in the utricle were quantified by Myo7a immunostaining. Compared with controls, the proportion of residual HCs was 72.22 ± 1.73%, 58.45 ± 2.47%, 46.40 ± 2.51%, 30.75 ± 2.66%, 21.49 ± 1.36%, and 6.40 ± 0.56% in the 2.5, 5, 10, 15, 20, and 25 ng/g DT groups, respectively (Fig. 1D and F). To precisely assess the HC loss in different regions, we counted HCs in the striola and the extrastriola separately (Fig. 1B). Compared with the controls, there were 73.89 ± 4.01%, 54.69 ± 2.03%, 40.05 ± 3.07%, 28.22 ± 2.88%, 20.00 ± 1.19%, and 6.37 ± 0.60% HCs remaining in the striola and 71.84 ± 1.83%, 59.32 ± 2.63%, 47.43 ± 2.78%, 31.34 ± 2.76%, 21.84 ± 1.46%, and 6.40 ± 0.57% HCs remaining in the extrastriola of the 2.5, 5, 10, 15, 20, and 25 ng/g DT groups, respectively (Fig. 1E and H). Linear regression analysis confirmed a strong negative correlation between DT dose and residual HC numbers (Fig. 1G), demonstrating that DT induces HC death in a dose-dependent manner.

### Both subtypes of utricular HCs were lost after DT treatment

Vestibular HCs are classified into type I and type II (Fig. 1C) [6, 7, 36–38]. To determine the differences in DT-induced damage to the two HC subtypes in the utricle, we assessed the molecular features of the residual HCs.

First, we identified type I and type II HCs by Sox2 and Myo7a immunostaining. In mature vestibular epithelium, Myo7a⁺/Sox2⁻ cells are considered type I HCs, while Myo7a⁺/Sox2⁺ cells represent type II HCs [39–41]. Quantification revealed that residual type I HCs (Myo7a⁺/Sox2⁻) in the 2.5, 5, 10, 15, 20, and 25 ng/g DT groups decreased to 98.23 ± 2.70%, 66.08 ± 5.60%, 44.03 ± 3.98%, 28.32 ± 6.53%, 24.48 ± 3.12%, and 2.36 ± 0.78% in the striola, and to 83.33 ± 5.19%, 77.60 ± 8.14%, 62.70 ± 0.67%, 30.27 ± 1.92%, 29.95 ± 3.76%, and 2.08 ± 0.69% in the extrastriola, respectively (Fig. 1I). By contrast, residual type II HCs (Myo7a⁺/Sox2⁺) decreased to 55.07 ± 0.44%, 36.12 ± 5.19%, 29.74 ± 6.57%, 17.84 ± 5.19%, 18.06 ± 1.17%, and 13.66 ± 1.17% in the striola, and to 59.61 ± 2.46%, 40.07 ± 2.46%, 37.87 ± 6.79%, 22.72 ± 4.38%, 15.64 ± 1.49%, and 13.03 ± 0.65% in the extrastriola, respectively (Fig. 1J).

**Fig. 2:**
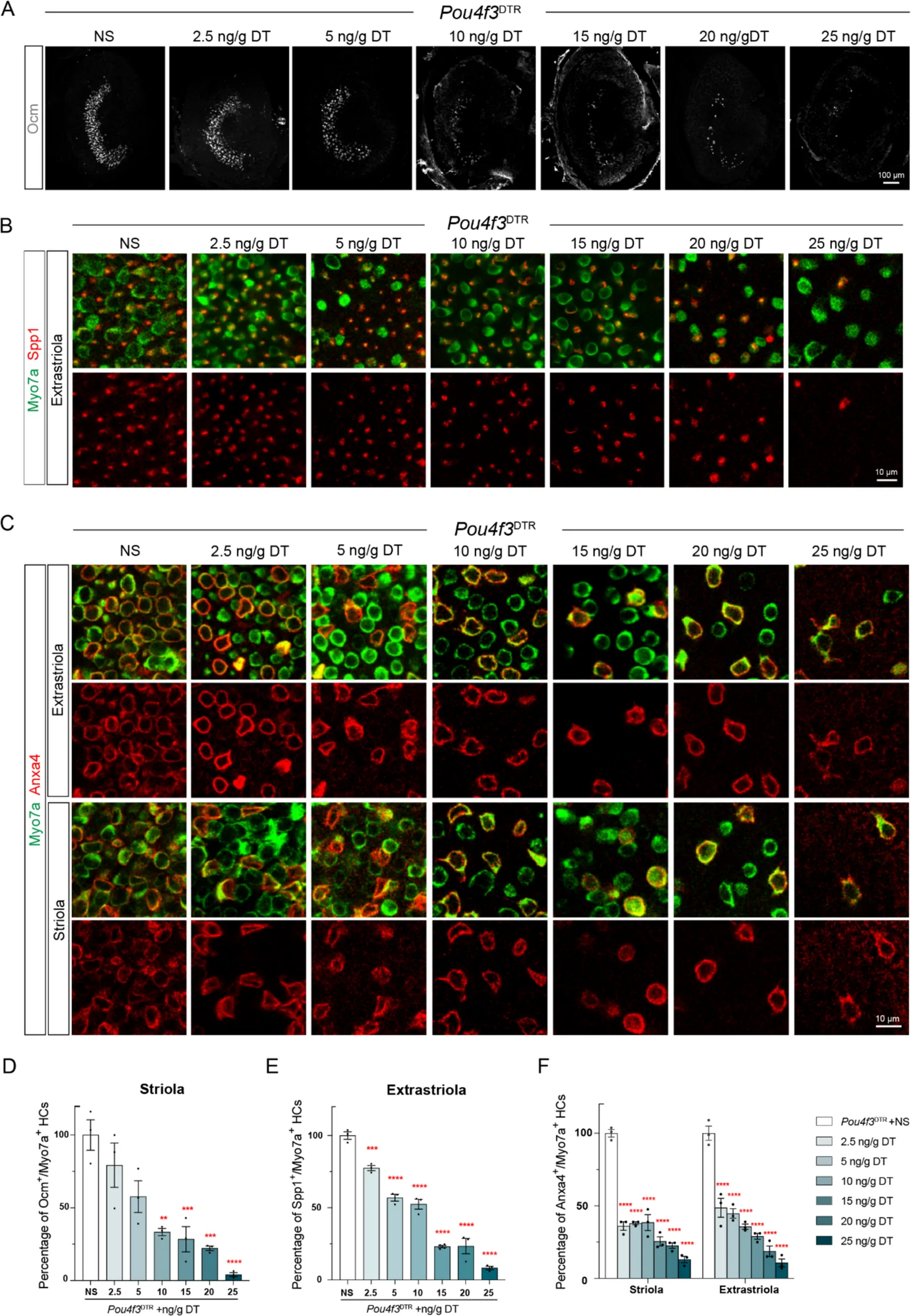
Both type I and type II utricular HCs decreased after DT treatment. (**A**) Low-magnification images showing a dose-dependent decrease in Ocm^+^ type I HCs in the striola (**B**) Representative images demonstrating a decrease in Spp1^+^/Myo7a^+^ type I HCs in the extrastriola following increasing doses of DT (**C**) Representative images demonstrating a decrease in Anxa4^+^/Myo7a^+^ type II HCs in both the striola and extrastriola following increasing doses of DT (**D-E**) Counts of Ocm^+^/Myo7a^+^ and Spp1^+^/Myo7a^+^ type I HCs expressed as percentages of the control group (*n* = 3 or 4) (**F**) Counts of Anxa4^+^/Myo7a^+^ Type II HCs expressed as percentages of the control group (*n* = 3) Scale bars: 100 μm in A; 10 μm in B and C. Red asterisks indicate statistical significance compared with the NS group. One-way ANOVA was performed for D-F. * *p* < 0.05, ** *p* < 0.01, *** *p* < 0.001, **** *p* < 0.0001

To validate these findings, we further labeled utricular HCs with other subtype markers: Ocm, Spp1 and Anxa4. Consistent with the Sox2/Myo7a results, the numbers of Myo7a⁺/Ocm⁺ striolar type I HCs (Fig. 2A and D), Myo7a⁺/Spp1⁺ extrastriolar type I HCs (Fig. 2B and E), and Myo7a⁺/Anxa4⁺ type II HCs (Fig. 2C and F) all declined progressively with increasing DT doses.

Together, these results demonstrate that both utricular HC subtypes are susceptible to DT in a dose-dependent manner.

### DT led to dose-dependent HC loss in the saccule and the crista

**Fig. 3:**
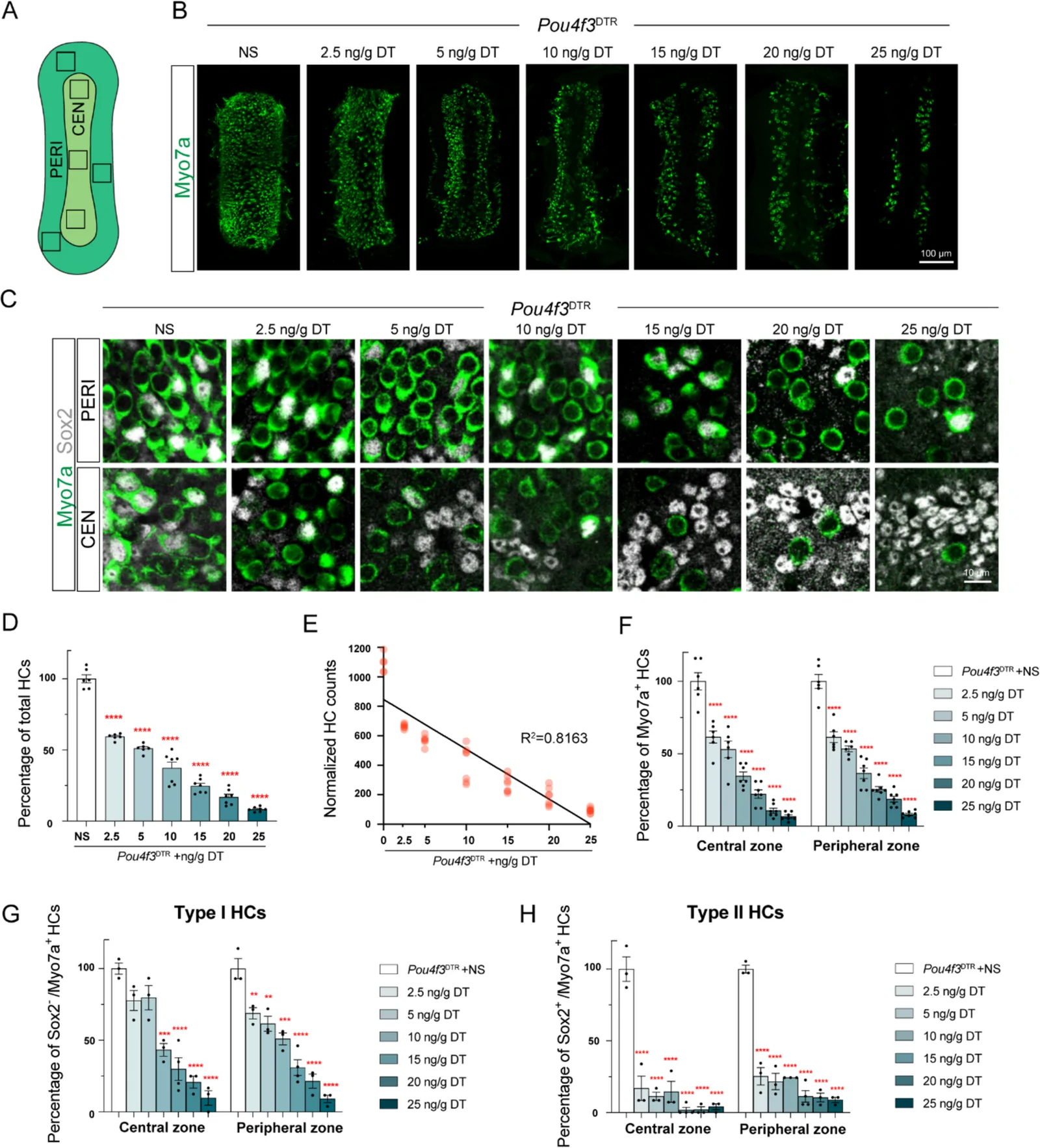
DT injection induced dose-dependent injury of crista HCs. (**A**) Schematic illustrating the central (CEN) and peripheral (PERI) zones of the horizontal cristae. Squares indicate sampling regions for quantitative statistics (**B**) Low-magnification images showing a decrease in Myo7a^+^ HC density with increasing doses of DT (**C**) Representative images demonstrating the reduction of Sox2^−^/Myo7a^+^ and Sox2^+^/Myo7a^+^ HCs in both the central and peripheral zones following increasing doses of DT (D) Total HC counts expressed as percentages of the control group (*n* = 6 or 7) (**E**) Numbers of residual crista HCs correlated with the doses of DT (simple linear regression, *p* < 0.0001) (**F-H**) Counts of Myo7a^+^ HCs (**F**) (*n* = 6 or 7), Sox2^–^/Myo7a^+^ type I HCs (**G**) (*n* = 3 or 4) and Sox2^+^/Myo7a^+^ type II HCs (**H**) (*n* = 3 or 4) in the central and peripheral zones expressed as percentages of the control group Scale bars: 100 μm in B; 10 μm in C. Red asterisks indicate statistical significance compared with the NS group. One-way ANOVA was performed for D, F-H. * *p* < 0.05, ** *p* < 0.01, *** *p* < 0.001, **** *p* < 0.0001

We next investigated the effect of DT on other vestibular organs of *Pou4f3*^DTR^ mice. For the crista, we chose the horizontal crista as representative (Fig. 3A). Total HC counts in the crista exhibited a dose-dependent decline with increasing DT doses (Fig. 3B, D and F). Both Myo7a⁺/Sox2⁻ type I HCs and Myo7a⁺/Sox2⁺ type II HCs showed a downward trend as the DT dose increased (Fig. 3C, G and H). In the saccule (Figure S1A), another otolith organ, the proportion of residual Myo7a^+^ HCs decreased dose-dependently from 56.69 ± 4.97% in the 2.5 ng/g DT group to 4.69 ± 0.47% in the 25 ng/g DT group (Figure S1B and S1D-S1F). Both type I (Myo7a⁺/Sox2⁻) and type II (Myo7a⁺/Sox2⁺) HCs in the striola and extrastriola declined in a pattern similar to that observed in the utricle (Figure S1C, S1G and S1H).

No consistent regional differences were observed within vestibular organs (Figure S2A-S2C). At lower DT concentrations (2.5 and 5 ng/g), type II HCs exhibited significantly lower survival than type I HCs (Figure S2D-S2I). However, as the DT dose increased and the overall number of surviving HCs declined, the difference in susceptibility between the two subtypes diminished.

Together, these findings indicate that DT-induced HC loss follows a similar dose-dependent pattern across all vestibular organs, including the utricle, saccule, and crista.

### The effect of DT treatment on stereociliary bundles and nerve innervation

**Fig. 4:**
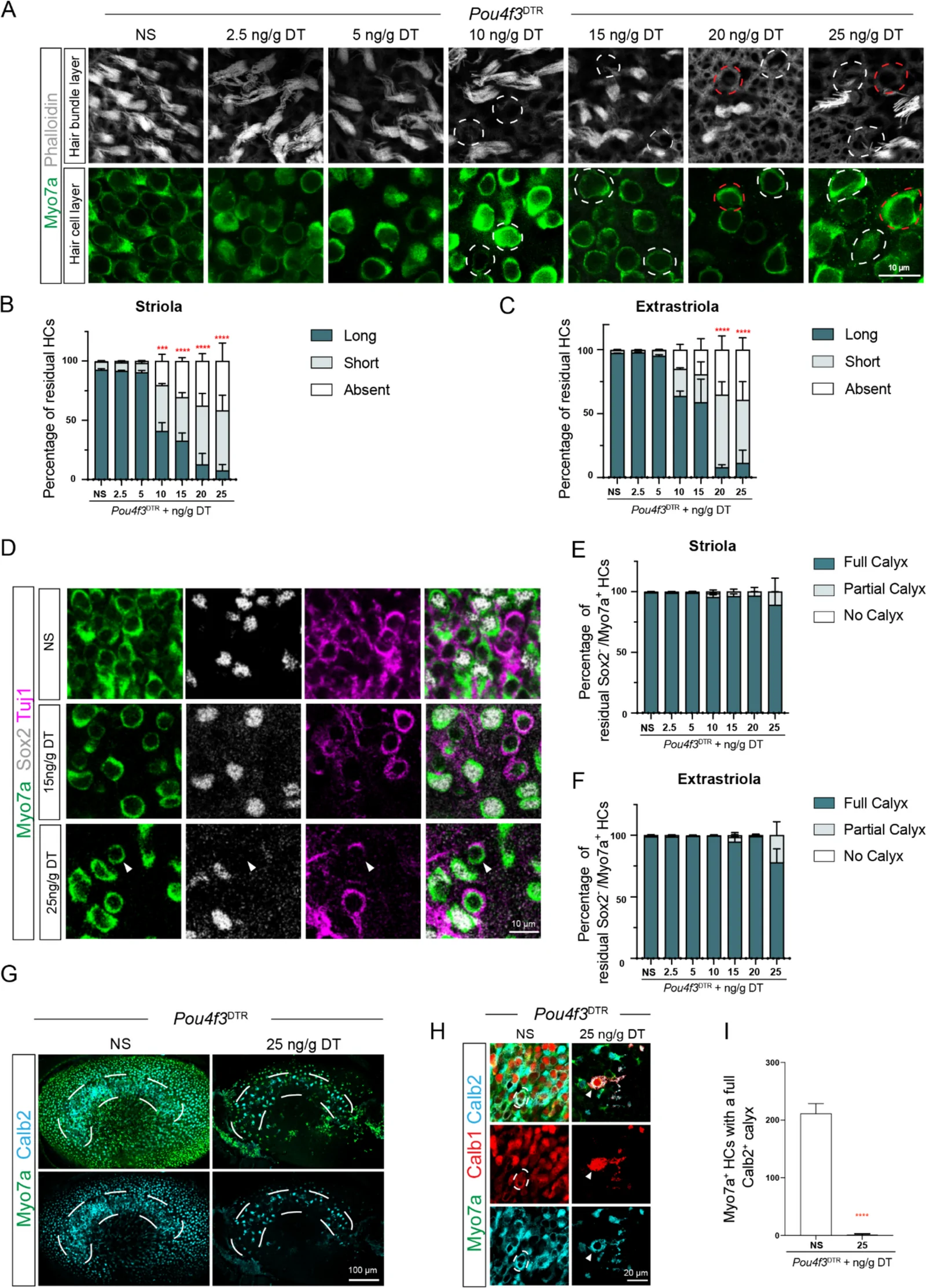
Stereociliary bundles and innervation of the residual utricular HCs after DT treatment. (**A**) High-magnification images of Phalloidin-labeled HC bundles. The white dashed lines mark HCs with short bundles, and the red dashed lines mark HCs with no bundles (**B-C**) Statistical data showing the percentages of residual HCs with long, short, or no bundles (*n* = 3). Statistical significance for the percentages of HCs with long bundles is indicated on the graph (**D**) Representative images showing Tuj1-labeled calyxes. The white arrowhead points to a HC with a partial calyx (**E-F**) Statistical data showing the percentages of residual type I HCs with full, partial, or no calyx. No significant differences were found in the percentages of residual type I HCs with full calyxes among these groups (*n* = 3) (**G**) Low-magnification images showing a decrease in Myo7a^+^ type I HCs with a full Calb2^+^ calyx after 25ng/g DT treatment. The white dashed lines delineate the boundaries of the striolar regions (**H**) Representative immunofluorescence images of Myo7a, Calb1, and Calb2. The white dashed lines mark HCs with a Calb2^+^ complex calyx. The white arrowhead points to the rare HC with a full Calb2^+^ calyx which is Calb1^+^ (**I**) Statistical data of the number of Myo7a^+^ HCs with a full Calb2^+^ calyx (*n* = 3) Scale bars: 10 μm in A and D; 100 μm in G; 20 μm in H. Red asterisks indicate statistical significance compared with the NS group. One-way ANOVA was performed for B, C, E, F. * *p* < 0.05, ** *p* < 0.01, *** *p* < 0.001, **** *p* < 0.0001

Then we examined other characteristics of residual HCs. Phalloidin labeling was used to assess stereociliary bundles in Myo7a⁺ HCs. In the control group, as well as in the 2.5 and 5 ng/g DT groups, most HCs exhibited long and well-structured bundles (Fig. 4A). However, starting from 10 ng/g DT, fewer HCs maintained long bundles, and at 25 ng/g DT, only 7.55 ± 5.25% of HCs in the striola and 11.34 ± 10.14% of HCs in the extrastriola retained long stereocilia (Fig. 4A and C), indicating progressive structural disruption with increasing DT dose.

To evaluate afferent innervation, we assessed nerve terminals surrounding type I HCs, focusing on the calyx endings, using Tuj1 immunostaining. The proportion of residual Myo7a⁺/Sox2⁻ type I HCs with full calyxes did not differ significantly among the control and DT-treated groups (Fig. 4D and F), although a few HCs with partial calyxes were noted at 25 ng/g DT group (Fig. 4D). Next, we stained for Calb2, which is primarily expressed in the extrastriolar type II HCs and nerve calyxes connecting type I HCs within the striola [42]. We observed that in the 25 ng/g DT group, Calb2 + calyxes were sparse (Fig. 4G and I), and the Calb2 + complex calyxes that wrap two or more striolar HCs were absent (Fig. 4H). Furthermore, the rare HCs with a Calb2 + calyx also expressed Calb1, a protein primarily expressed in calyxes and type I HCs within the striola [42] (Fig. 4H). This suggests that DT treatment did not severely damage the nerve terminals. We further assessed synaptic integrity by labeling pre- and post-synaptic markers, Ctbp2 and Shank1/2/3. Quantification revealed no significant differences in either paired synapses or orphan Ctbp2⁺ puncta among the control, 15 ng/g DT, and 25 ng/g DT groups (Figure S3A and S3B). These results indicate that, despite severe HC loss at high DT doses, the synaptic structures of surviving HCs remain largely intact.

### The dependence of vestibular function on HC number

Human vestibular function is assessed by balance function tests and eye movement recordings following visual stimulation, positional changes, thermal or rotational stimuli [43]. However, due to the differences in body size, the strategies employed for mice differ from those used for humans. When the central nervous system and motor system are intact, balance function tests, such as the open field test, the swimming test, tail-hanging and air-righting reflexes are widely used to assess the vestibular function [14, 23, 44–46]. More precise measurements can be obtained using horizontal angular vestibulo-ocular reflex (HAVOR) tests to assess crista function, and off-vertical axis rotation (OVAR) tests to evaluate utricular function [47, 48].

**Fig. 5:**
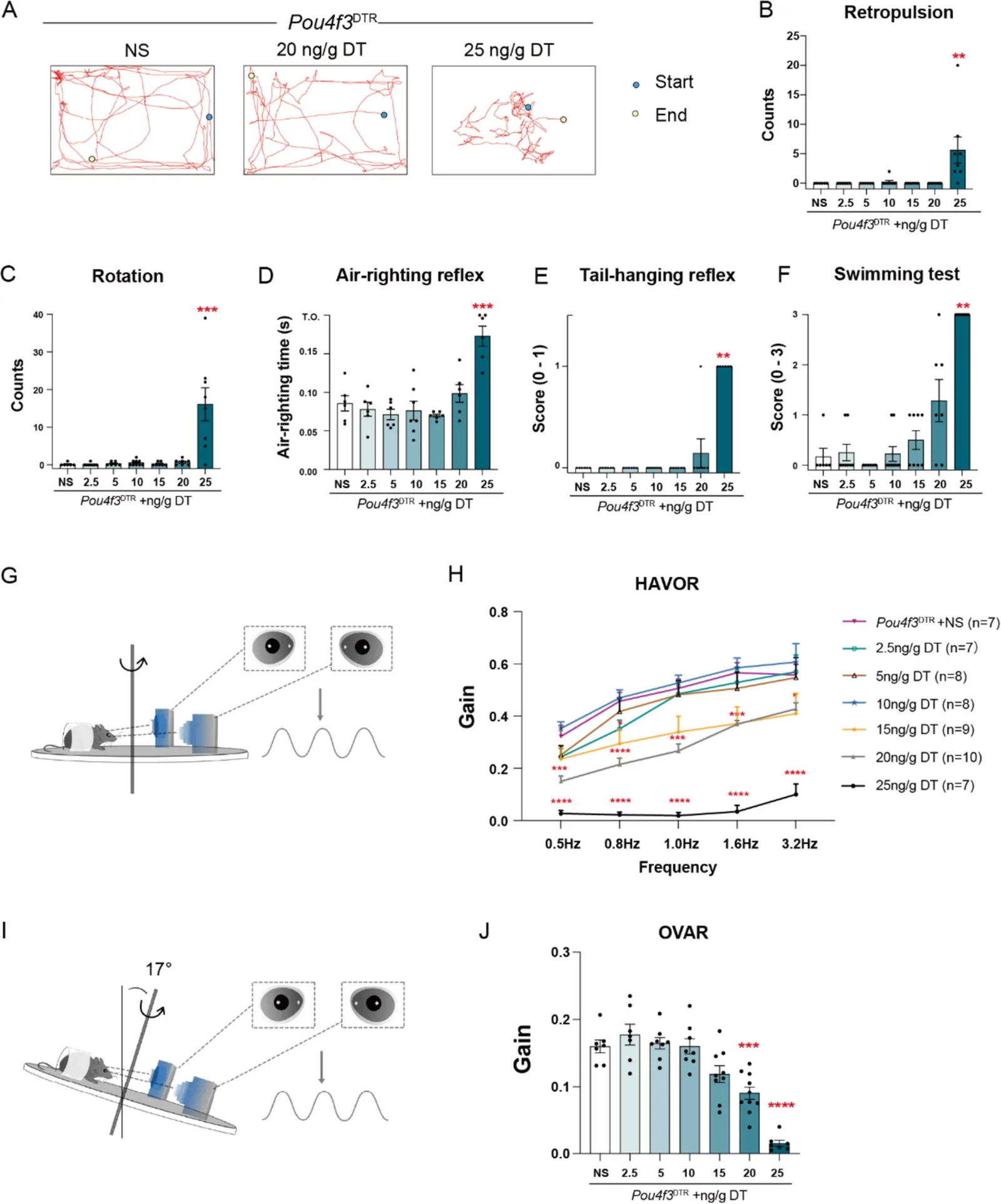
Vestibular function was affected after DT treatment. (**A**) Representative pathway traces from the open field tests of *Pou4f3*^DTR^ mice receiving NS, 20 ng/g DT, or 25 ng/g DT (**B-C**) Quantification of retropulsion and rotation events in the open field tests (*n* = 6, 7, 8, or 9) (**D**) Quantification of the air-righting time (*n* = 6 or 7) (**E-F**) Quantification of the scores for mouse behaviors in the tail-hanging reflex (**E**) and swimming test (**F**) (*n* = 6, 7, 8, or 9) (**G-H**) Schematic and functional results of the HAVOR tests (*n* = 7, 8, 9, or 10) (**I-J**) Schematic and functional results of the OVAR tests (*n* = 7, 8, 9, or 10) Red asterisks indicate statistical significance compared with the NS group. One-way ANOVA was performed for B-D, H and J. Mann-Whitney test was performed for E and F. * *p* < 0.05, ** *p* < 0.01, *** *p* < 0.001, **** *p* < 0.0001

We explored the relationship between vestibular function and HC number using the DT-induced dose-dependent injury model. Balance function tests were first used to evaluate gross vestibular function. In the open field test, control mice and mice injected with ≤ 20 ng/g DT explored mainly along the edges of the field in straight trajectories with few retropulsion and rotation events. In contrast, mice received 25 ng/g DT moved mostly in the center along curved paths (Fig. 5A, video 1), and displayed 5.63 ± 2.23 retropulsion and 16.13 ± 4.39 rotation events (Fig. 5B and C), indicating severe vestibular impairment. In the air-righting reflex test, no significant differences were observed in the 2.5, 5, 10, 15, and 20 ng/g DT groups compared to controls. However, the air-righting time was prolonged in 25 ng/g DT mice (0.17 ± 0.01 s vs. 0.09 ± 0.01 s in controls; Fig. 5D, video 2). In the tail-hanging tests, the mice in the 2.5, 5, 10, and 15 ng/g DT groups behaved similarly to those in the control groups, tending to extend their forelimbs (score 0), while 1 of the 7 mice in the 20 ng/g DT group and all mice in the 25 ng/g DT group ventrally bent their bodies (score 1) (Fig. 5E). In the swimming test, when the DT dose was less than 20 ng/g, most of the mice could swim normally (score 0). However, in the 20 ng/g DT group, some mice displayed immobile floating (2 of 7, score 2), or struggled in the water (1 of 7, score 3), and in the 25 ng/g DT group, all mice were unable to swim (score 3; Fig. 5F, video 3). Taken together, these tests indicated that the residual HCs in the 15 ng/g DT group were sufficient to maintain balance function, corresponding to 30.75 ± 2.66% (utricle), 17.23 ± 3.13% (saccule), and 24.67 ± 1.92% (crista) of control HC counts (Figs. 1F and 3D and S1D).

To more precisely evaluate the vestibular function, we used the HAVOR and OVAR tests. Eye movements were tracked to calculate gain values (Fig. 5G and I). In the control group, HAVOR gain values were 0.32 ± 0.04, 0.46 ± 0.03, 0.51 ± 0.04, 0.57 ± 0.04, and 0.56 ± 0.04 at 0.5, 0.8, 1.0, 1.6, and 3.2 Hz, respectively. HAVOR gains significantly decreased in the 15 ng/g (0.8 Hz), 20 ng/g (all frequencies), and 25 ng/g groups (all frequencies; Fig. 5H; Figure S4). In the 25 ng/g group, gain values dropped to 0.03 ± 0.01, 0.02 ± 0.01, 0.02 ± 0.01, 0.03 ± 0.02, and 0.10 ± 0.04 at 0.5, 0.8, 1.0, 1.6, and 3.2 Hz, respectively (Fig. 5H; Figure S4, video 4). The OVAR gain value was 0.16 ± 0.01 in the control group and was reduced significantly in the 20 ng/g and 25 ng/g DT groups, which were 0.09 ± 0.01 and 0.02 ± 0.00, respectively (Fig. 5J, video 5). These results indicate that the residual HCs in the 10 ng/g DT group were sufficient to maintain normal vestibulo-ocular reflexes, corresponding to 46.40 ± 2.51% (utricle), 37.42 ± 2.33% (saccule), and 37.25 ± 4.12% (crista) of control HC counts (Figs. 1F and 3D; Figure S1D).

### The contribution of HC subtype to vestibular function

Based on the above results, mice treated with 25 ng/g DT exhibited significantly greater vestibular impairment than those receiving 15 ng/g DT (Fig. 5A and J). Interestingly, the number of type II HCs was comparable between these two groups, whereas type I HCs were markedly reduced in the 25 ng/g DT group (Figure S5A), suggesting a critical role for type I HCs in maintaining vestibular function.

To test this hypothesis, we employed two mouse models with selective type I HC depletion or phenotypic conversion. The first model was based on low-dose IDPN administration, which preferentially eliminates type I HCs [14]. The second was the *Gfi1*^Cre^/*Atoh1*-OE transgenic mouse model, in which *Atoh1* overexpression in HCs reprograms most type I vestibular HCs into type II-like cells [8].

**Fig. 6:**
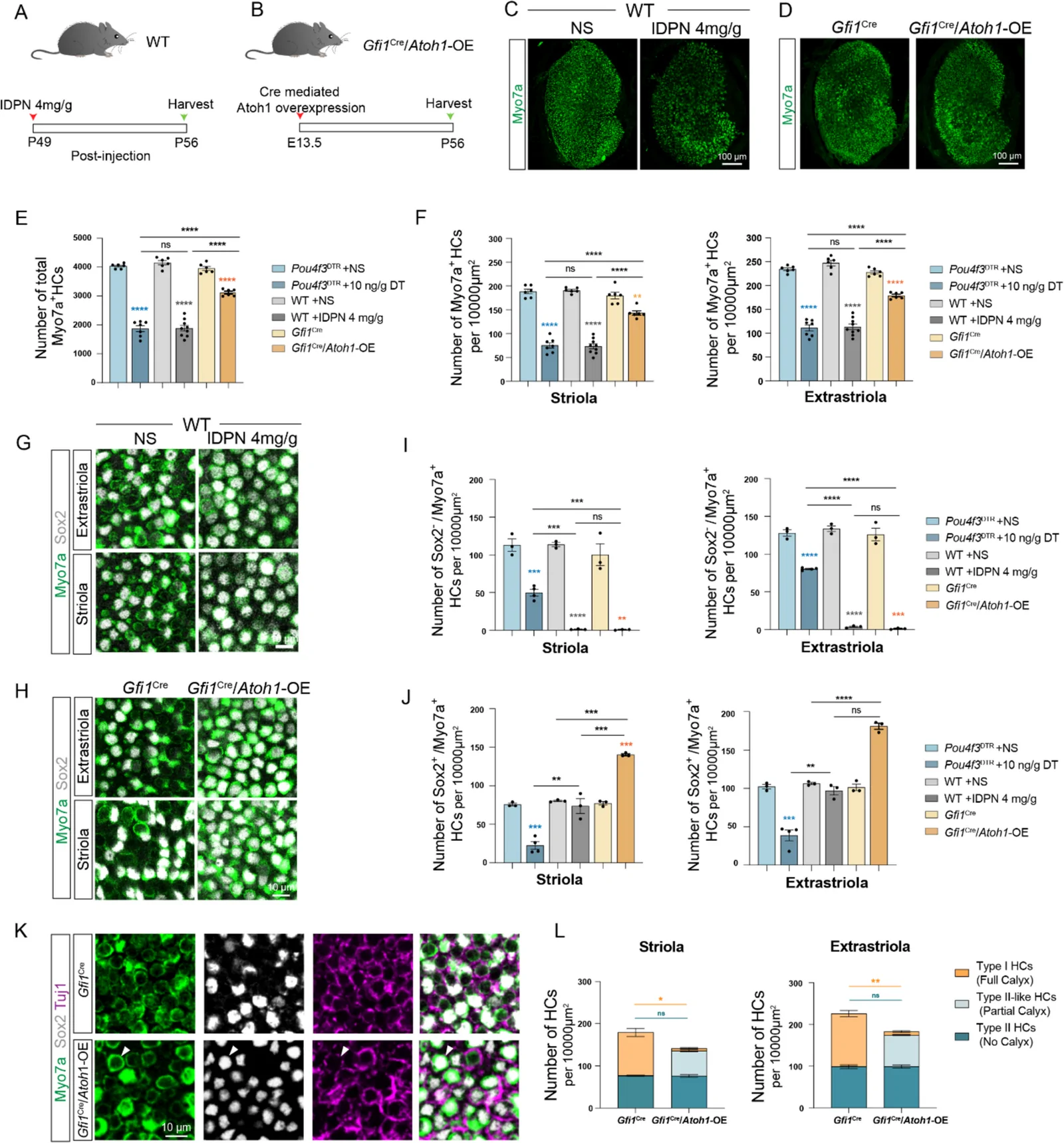
IDPN injection or ***Atoh1*** overexpression reduced the number of utricular type I HCs (**A-B**) Schematic illustration of the experiments. WT mice received NS or 4 mg/g IDPN at P49, and utricles were harvested 1 week later (**A**). *Gfi1*^Cre^ mice and *Gfi1*^Cre^/*Atoh1*-OE mice were harvested at P56 (B) (**C-D**) Low-magnification images showing Myo7a^+^ HC density in the utricles (**E-F**) Quantification of utricular HC numbers. The HC numbers in the 10 ng/g DT group were comparable to those in the IDPN group but lower than those in the *Gfi1*^Cre^/*Atoh1*-OE group (*n* = 6, 7, 8, or 9) (G-H) Representative images demonstrating that most utricular HCs in IDPN-treated mice (**G**) or *Gfi1*^Cre^/*Atoh1*-OE mice (**H**) were Sox2^+^ HCs in both the striola and extrastriola (**I-J**) Quantification of Sox2^–^/Myo7a^+^ HCs (I) and Sox2^+^/Myo7a^+^ HCs (J) in the striola and extrastriola (*n* = 3 or 4) (**K**) Representative immunofluorescence images of Myo7a, Sox2, and Tuj1, showing the Tuj1⁺ nerve terminals innervating HCs. The white arrowhead points to a HC with a partial calyx (**L**) Classification of HC types by Tuj1⁺ calyx morphology (Type I, Type II-like, Type II). A significant reduction was observed in the number of type I HCs (orange asterisks), whereas the number of type II HCs showed no significant change (*n* = 3) Scale bars: 100 μm in C and D; 10 μm in G, H and K. Blue, grey, and orange asterisks (in E, F, I and J) indicate statistical significance compared with the respective controls. Black asterisks indicate statistical significance between the two groups marked by the straight lines. Data were analyzed by unpaired t-test. * *p* < 0.05, ** *p* < 0.01, *** *p* < 0.001, **** *p* < 0.0001, ns, not significant

**Fig. 7:**
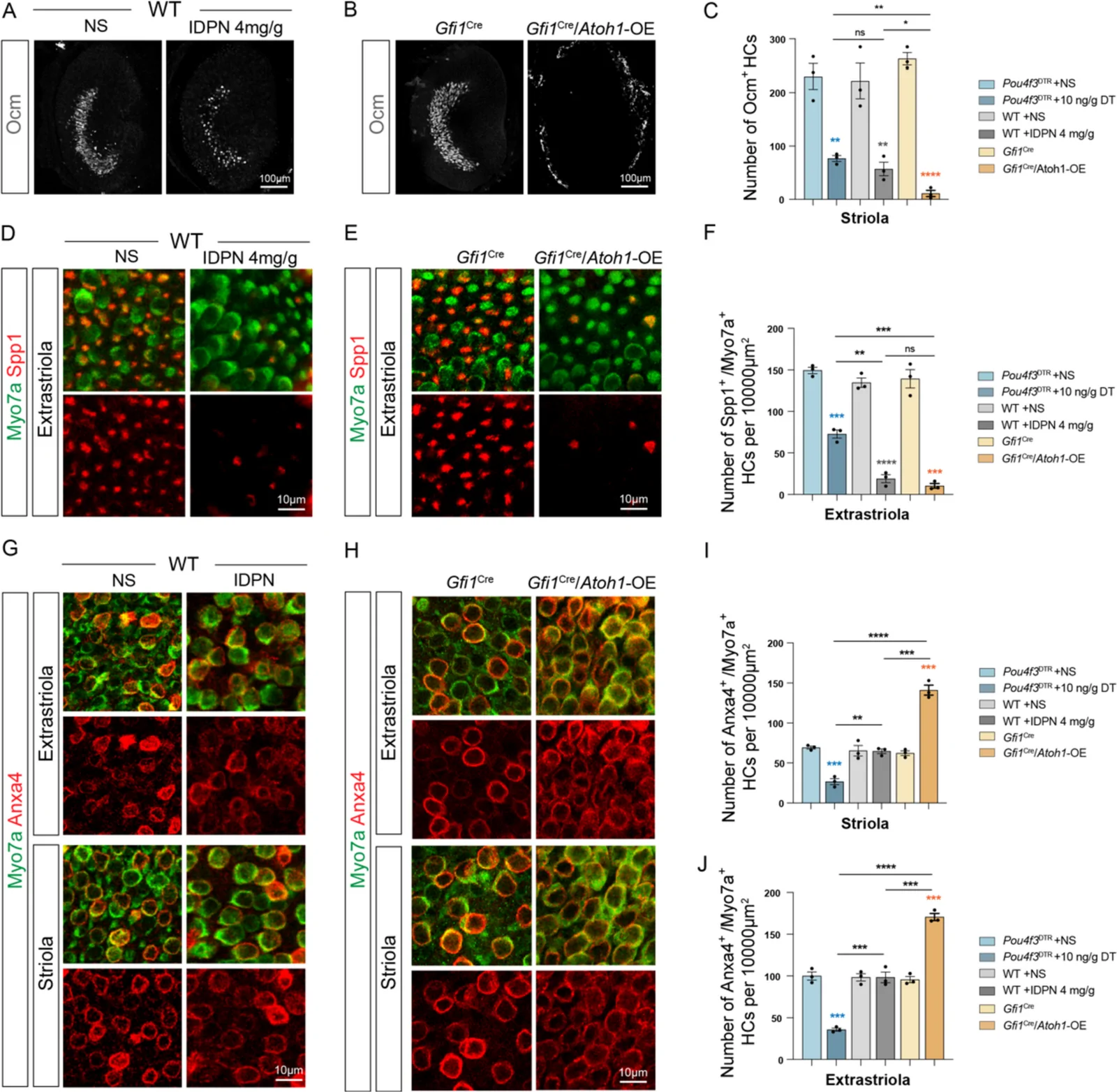
Characterization of subtype markers in utricular HCs of IDPN-treated or ***Atoh1*** overexpression mice (**A-B**) Confocal images showed a decrease in Ocm^+^ type I HCs in the striola after IDPN treatment or *Atoh1* overexpression (**C**) Quantification of Myo7a^+^/Ocm^+^ type I HCs in the utricular striola (*n* = 3) (**D-E**) Confocal images showed a decrease in Spp1^+^ type I HCs in the extrastriola after IDPN treatment or *Atoh1* overexpression (**F**) Quantification of Myo7a^+^/Spp1^+^ type I HCs in the utricular extrastriola (*n* = 3) (**G-H**) Co-immunofluorescence imaging for Myo7a and Anxa4 in the extrastriola and striola of different groups (**I-J**) Quantification of Myo7a^+^/Anxa4^+^cells (*n* = 3) Scale bars: 100 μm in A-B; 10 μm in D, E, G and H. Blue, grey, and orange asterisks indicate statistical significance compared with the respective controls. Black asterisks indicate statistical significance between the two groups marked by the straight lines. Unpaired t-test was performed for C, F, I, J. * *p* < 0.05, ** *p* < 0.01, *** *p* < 0.001, **** *p* < 0.0001, ns, not significant

**Fig. 8:**
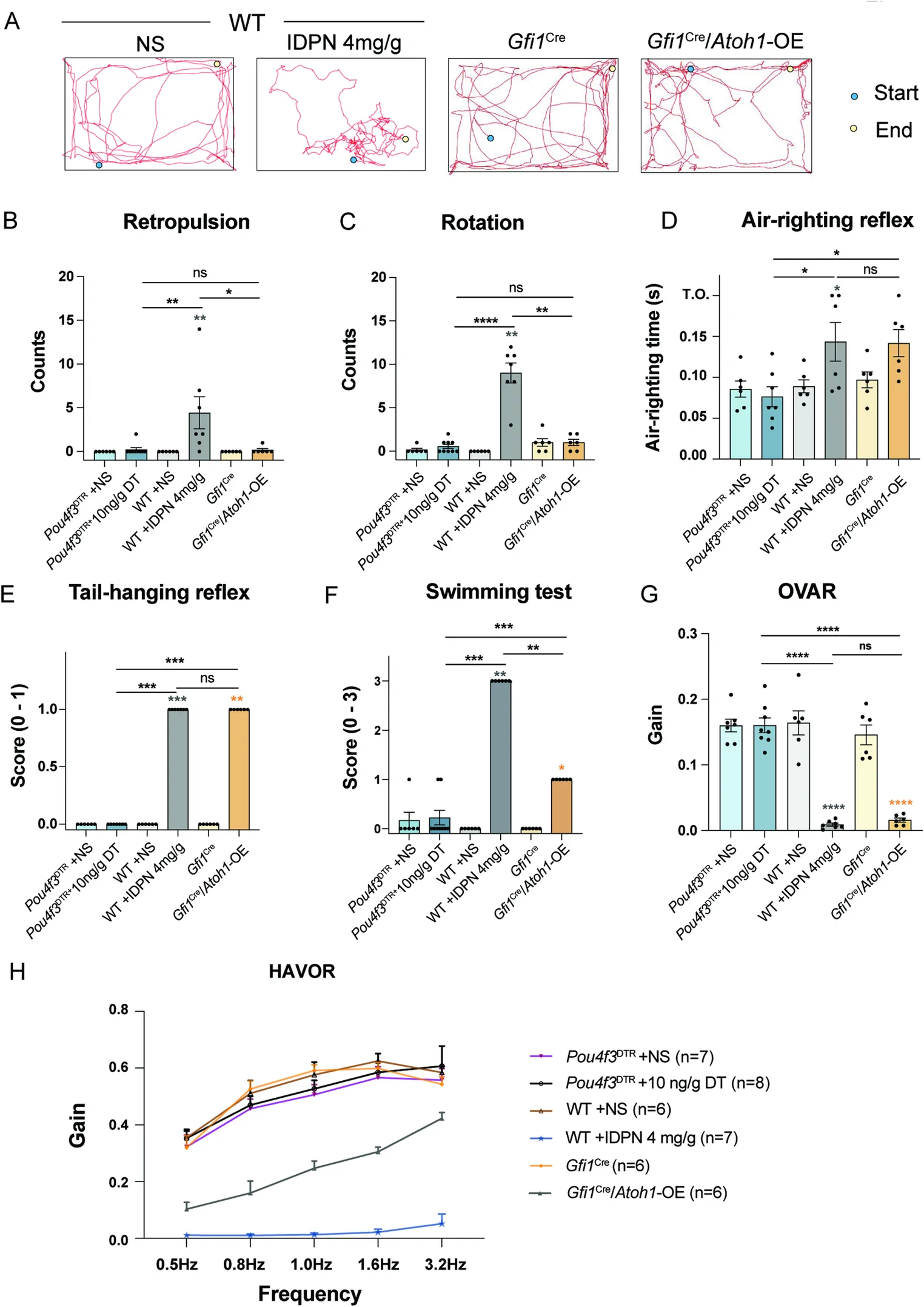
Vestibular function was more severely impaired in type I HC-deficient mice compared with 10 ng/g DT-injected ***Pou4f3***^DTR^ mice (**A**) Representative pathway traces from the open field tests (**B-C**) Quantification of retropulsion (**B**) and the rotation (**C**) events in the open field tests (*n* = 6, 7, 8 or 9) (**D**) Quantification of the air-righting time (*n* = 6 or 7) (**E-F**) Quantification of the scores for mouse behaviors in the tail-hanging reflex (**E**) and swimming test (**F**) (*n* = 6, 7, 8, or 9) (**G**) Quantification of OVAR gain values (*n* = 6, 7, or 8) (**H**) Quantification of HAVOR gain values (*n* = 6, 7, or 8). The corresponding statistical analysis is shown in Figure S9 Blue, grey, and orange asterisks indicate statistical significance compared with the respective controls. Black asterisks indicate statistical significance between the two groups marked by the straight lines. Unpaired t-test was performed for B-D, G and H; Mann-Whitney test was performed for E and F. * *p* < 0.05, ** *p* < 0.01, *** *p* < 0.001, **** *p* < 0.0001

In the IDPN model, wild-type mice were injected with 4 mg/g IDPN. As acute HC loss is typically assessed 7 days after IDPN injection [14, 16, 23], we used 7-week-old mice to ensure analysis at age 8 weeks, matching the *Pou4f3*^DTR^–DT model (Fig. 6A). The numbers of residual utricular HCs (Fig. 6C, E and G), crista HCs (Figure S7A, S7C, S7D and S7F) and saccular HCs (Figure S8A and S8C-S8E) in the IDPN group were comparable to those in the 10 ng/g DT group. We then compared vestibular HC subtypes, hair bundle morphology, neural innervation, and vestibular function between these two groups. In IDPN-treated mice, Sox2⁻/Myo7a⁺ type I HCs were selectively lost, leaving predominantly Sox2⁺/Myo7a⁺ type II HCs (Fig. 6G, I and J; Figures S7D, S7G, S7H, S8E, S8G and S8H). This selective loss of type I HCs was further confirmed by Ocm, Spp1, and Anxa4 immunostaining (Fig. 7A and J). We note that although Sox2-negative type I HCs in the striola were nearly absent following IDPN treatment, a small number of Ocm-positive cells remained (Fig. 7A). This may reflect previously reported non-specificity of Ocm, which also labels a proportion of type II HCs [49].Compared to the 10 ng/g DT group, the vestibular epithelia of IDPN-treated mice contained significantly less type I HCs and more type II HCs. The proportion of utricular HCs with long hair bundles was similar between the two groups (Figure S6A and S6B). Behaviorally, IDPN-treated mice exhibited profound balance deficits in the open field, tail-hanging, air-righting, and swimming tests (Fig. 8A and F). Vestibulo-ocular reflexes (HAVOR and OVAR) were also nearly abolished in this group (Fig. 8G H). In contrast, *Pou4f3*^DTR^ mice treated with 10 ng/g DT retained normal vestibular function despite having a comparable total HC count but significantly more type I HCs (Fig. 8A H, 6E, 6F, 6I and 6J). These findings support the notion that type I HCs are essential for vestibular function.

In the *Gfi1*^Cre^/*Atoh1*-OE model, our previous study [8] showed that by P35, total vestibular HC numbers were comparable to those of controls, although presumptive type I HCs had undergone conversion into type II-like HCs. In the current study, we evaluated vestibular HCs and function at P56 to allow direct comparison with other models (Fig. 6B). In the utricle (Fig. 6D, F and H), the crista (Figure S7B, S7C, S7E, S7F) and the saccule (Figure S8B-S8D, S8F), we observed a reduction in HC numbers, indicating some degree of HC loss. Immunofluorescence analysis with subtype markers revealed that, in the *Gfi1*^Cre^/*Atoh1*-OE group, Sox2^–^, Spp1^+^, or Ocm^+^ type I HCs (Figs. 6H and I and 7B C, 7E and 7 F) were nearly absent in the utricular epithelium, with almost all HCs being Sox2^+^ or Anxa4^+^ (Fig. 6H and J H-7J). Similar phenotypes were observed in the crista (Figure S7E, S7G, S7H) and saccule (Figure S8F-S8H). Based on cell morphology and Tuj1 + nerve terminals, Myo7a^+^ HCs in the *Gfi1*^Cre^/*Atoh1*-OE mice were categorized into three distinct types: Type I (full calyx), Type II-like (partial calyx), and Type II (no calyx). Cell counts showed that the number of utricular type II HCs in the *Gfi1*^Cre^/*Atoh1*-OE group was comparable to controls, indicating that *Atoh1* overexpression did not affect type II HC numbers. Instead, it led to a reduction in type I HCs and induced their phenotypic conversion (Fig. 6K and L). Compared with the 10 ng/g DT group, *Gfi1*^Cre^/*Atoh1*-OE mice exhibited fewer type I HCs, a comparable number of type II HCs, and a higher proportion of HCs with long stereocilia (Figure S6A and S6B). Functionally, *Gfi1*^Cre^/*Atoh1*-OE mice performed normally in the open field test, but showed significant impairments in tail-hanging, air-righting, swimming, HAVOR, and OVAR tests (Fig. 8A H; S9A-S9E). In comparison, despite having fewer total HCs, the 10 ng/g DT group (*Pou4f3*^DTR^ mice) retained more type I HCs and exhibited superior vestibular performance (Fig. 6E, F, I and J H). Collectively, these results demonstrate that type I HCs are indispensable to maintain balance and vestibulo-ocular reflexes.

## Discussion

An effective and well-characterized vestibular HC injury model is crucial for studying HC function, regeneration, and the mechanisms underlying vestibular dysfunction. Traditional vestibulotoxic drugs—such as gentamicin, cisplatin, and IDPN—have been widely used to damage the vestibular sensory epithelium. However, these models have notable limitations, including low selectivity for HCs and high animal mortality due to systemic toxicity [20, 22, 50–52]. In this study, we improved the vestibular HC injury model using *Pou4f3*^DTR^ transgenic mice with DT administration. Increasing DT doses from 2.5 ng/g to 25 ng/g induced progressive, dose-dependent vestibular HC loss. Importantly, this HC-specific ablation occurred across vestibular subregions (central vs. peripheral; striolar vs. extrastriolar) and HC subtypes. When the DT dose reached 10 ng/g, the stereocilia of the remaining utricular HCs began to undergo degenerative changes, and when the DT dose reached the highest dose of 25 ng/g, the nerve endings of the remaining HCs in the utricle began to show slight but not statistically significant damage (Fig. 4D and F). These results indicated that the *Pou4f3*^DTR^-DT adult mouse vestibular injury model was a dose-dependent HC-specific injury model and produced relatively uniform damage across HC subtypes.

Vestibular organs are involved in controlling body balance through multiple reflexes, including the vestibulo-spinal, vestibulo-ocular, and vestibulo-neck reflexes. Because *Pou4f3* is exclusively expressed in inner ear HCs, DT administration in this model does not damage vestibular ganglion neurons or central nervous system structures, making it ideal for examining the dependence of vestibular function on HC number. Balance function experiments showed that at ≤ 15 ng/g DT, mice exhibited no significant deficits in open-field, anti-gravity reflex, or swimming tests. When the dose was ≤ 10 ng/g, mice also performed normally in vestibulo-ocular reflex tests (HAVOR and OVAR). Correlating these functional outcomes with residual HC numbers revealed that balance function remained largely intact with ~ 30% of HCs, and vestibular-ocular reflexes were preserved with ~ 50% residual HCs. These results suggest that mouse bilateral vestibular function is not very sensitive to the partial loss of vestibular HCs. Clinically, patients undergoing cisplatin chemotherapy often experience hearing loss and tinnitus, yet vestibular dysfunction is less frequently reported [53]. This discrepancy may reflect the functional redundancy of vestibular HCs to balance maintenance and vestibular-ocular reflexes, as well as differential susceptibility of cochlear and vestibular HCs to cisplatin and potential central vestibular compensation known to occur in humans. On the other hand, our findings also suggest that current behavioral assays may lack the sensitivity to detect subtle vestibular deficits in mice with > 50% residual HCs, highlighting the need for more refined testing methods.

Ciliary bundles on the apical surface of HCs are critical for mechanoelectrical transduction. Severe bundle abnormalities or complete loss abolish this function, leading to vestibular deficits [4, 54, 55]. Interestingly, mild stereociliary defects may be functionally tolerated. For example, Brg1-deficient mice display fused stereocilia without HC loss and maintain normal balance, suggesting a threshold for functional tolerance [56]. Consistent with this, in our study, ~ 97% of HCs in control mice had long hair bundles, whereas in the 10 ng/g DT group, only 40.90 ± 7.21% (striola) and 63.77 ± 3.87% (extrastriola) HCs retained intact bundles. Despite this, mouse vestibular function in the 10 ng/g DT group remained normal, implying that limited stereociliary damage does not critically compromise function and that structural redundancy may exist in adult vestibular HCs.

The specific roles of type I and type II HCs in vestibular function are not fully understood. Our data provide compelling evidence that type I HCs are essential to maintain vestibular function. Three lines of evidence support this conclusion. First, mice receiving 25 ng/g DT exhibited severe vestibular deficits relative to the 15 ng/g group, despite similar type II HC numbers, attributable to greater type I HC loss. Second, IDPN-treated mice exhibited profound functional impairments, whereas the 10 ng/g DT group maintained normal function. Although both groups had similar total HC numbers, IDPN mice retained primarily type II HCs with far fewer type I HCs. Third, in *Gfi1*^Cre^/*Atoh1*-OE mice, reprogramming type I HCs into type II–like cells led to vestibular dysfunction despite type II HC numbers and morphology being comparable to controls. Together, these findings indicate that the preservation of type I HCs is critical for normal vestibular performance.

We acknowledge that this study has several limitations. In the Pou4f3^DTR^ model, the 10 ng/g DT dose significantly reduced both type I and type II HCs, precluding us from ruling out a cumulative contribution of both subtypes to the observed behavioral deficits. In the IDPN model, it remains unclear whether afferent innervation is directly affected, which could confound interpretation of functional specificity. The Atoh1 overexpression model, as a developmental mutant, may also introduce pleiotropic effects on neuronal development that are not yet fully characterized. Future studies should aim to identify subtype-specific vestibular HC markers not expressed in central vestibular pathways or vestibular ganglion neurons, and to develop Cre-mediated, cell type–specific ablation models for directly dissecting the functional relevance of distinct HC subtypes.

In mature mammalian vestibular organs, spontaneous HC regeneration is insufficient to restore function after injury. Strategies such as manipulating Notch and Wnt signaling or overexpressing *Atoh1* in supporting cells can promote HC regeneration and modest functional recovery, primarily yielding type II HCs [16, 18, 57]. For instance, *Atoh1* overexpression restored 80.4% of utricular HCs after IDPN injury; however, type I HCs remained scarce, and functional recovery was minimal (limited to vestibular evoked potentials) [16]. In contrast, our findings demonstrate that even with only ~ 50% remaining HCs, balance function and vestibular-ocular reflexes can be fully maintained if HC subtype composition and neural connectivity are preserved. Moreover, recent studies indicate that repair of type I HC afferent innervation is closely associated with functional recovery [58]. These observations emphasize that increasing total HC numbers alone may be insufficient; ensuring appropriate subtype ratios and afferent connectivity is essential for meaningful functional regeneration. Future strategies should therefore prioritize generating or converting type I HCs and restoring their neural connections.

In conclusion, we have established a selective, dose-dependent vestibular HC injury model and systematically evaluated how vestibular function depends on HC number and subtype. By integrating results from three distinct injury models, we demonstrate functional redundancy among vestibular HCs and highlight the indispensable role of type I HCs. These findings provide critical insights for the development of regenerative therapies aimed at restoring vestibular function and suggest that future interventions should prioritize HC quality—particularly subtype identity and neural connectivity—over merely HC quantity.

## Materials and methods

### Animals and genotyping

*Pou4f3* ^DTR^ mice were purchased from the Jackson Laboratory (Stock 028673). *Gfi1*^Cre^ and CAG-loxP-stop-loxP-*Atoh1*-HA (*Atoh1*-OE) mice were kind gifts from the University of Rochester and St. Jude Children’s Research Hospital. Mice of both sexes were used. The mating and genotyping of *Gfi1*^Cre^/*Atoh1*-OE and *Pou4f3*^DTR^ mice were performed as described in previous studies [8, 29, 35]. Mice were maintained in a quiet room with free access to food and water. All animal procedures were approved by the Institutional Animal Care and Use Committee at Fudan University and adhered to the National Institute of Health’s Guide for the Care and Use of Laboratory Animals. The experimental protocol was approved by the Animal Ethics Committee of Fudan University (approval code: 202107010 S; approval date: 22 July 2021).

### HC ablation

For DT-mediated HC ablation, adult *Pou4f3*^DTR^ mice (6 weeks old) received two intramuscular DT injections (2.5–50 ng/g; List Biological Laboratories) 48 h apart, with tissue collection 14 days post-initial injection. For IDPN-mediated HC ablation, wild-type mice (7 weeks old) received a single IDPN injection (4 mg/g; TCI Shanghai, Cat. No. 10010), and inner ear samples were collected 7 days later.

### Vestibular function tests

Mouse balance function was evaluated using a series of behavioral tests, including the tail-hanging reflex, air-righting reflex, open-field test, and swimming test [35, 45, 46]. Detailed experimental procedures and scoring criteria were as described in our established protocols [35].

Mouse vestibulo-ocular reflex was assessed using a binocular video-oculography system (Gianttek, GAT-MVOR943), following our established protocols [35]. Briefly, for the horizontal angular vestibulo-ocular reflex (HAVOR, also known as aVOR) test [59], mice were secured on a rotating platform and subjected to yaw (horizontal) sinusoidal rotations at frequencies of 0.5, 0.8, 1.0, 1.6, and 3.2 Hz. Eye movements were recorded synchronously using infrared cameras. In the off-vertical axis rotation (OVAR) test, mice were fixed in a nose-down position on a platform tilted 17° from the horizontal plane [44, 48, 59]. Gain values, defined as the ratio of the response amplitude to the stimulus amplitude, were calculated from the eye movement data.

### Immunohistochemistry

After microdissection, the utricles, saccules, and cristae were fixed in 4% paraformaldehyde at room temperature for 1 h. The tissues were then rinsed three times with PBS and were blocked with 10% donkey serum (with 1% Triton X-100 in 0.01 M PBS) for 12 h at 4 °C and then incubated in diluted primary antibodies overnight at 4 °C. After washing with PBS, the tissues were incubated with diluted secondary antibodies for 12 h at 4 °C. The following antibodies were used: anti-Myo7a (1:500 dilution, Proteus BioSciences, Cat# a25-6790, RRID: AB_2314838), anti-Myo7a (1:100 dilution, Santa Cruz Biotechnology, Cat# sc-74516, RRID: AB_2148626), anti-Sox2 (1:300 dilution, Santa Cruz Biotechnology, Cat# SC17319, RRID: AB_661259), anti-Tuj1 (1:300 dilution, BioLegend, Cat# 801202, RRID: AB_2313773), anti-Annexin A4 (anti-Anxa4, 1:200 dilution, R and D Systems, Cat# AF4146, RRID: AB_2242796), anti-Oncomodulin (anti-Ocm, 1:300 dilution, Santa Cruz Biotechnology, Cat# sc-7446, RRID: AB_2267583), anti-Osteopontin (anti-Spp1, 1:200 dilution, R and D Systems, Cat# AF808, RRID: AB_2194992), anti-Calb2 (1:200 dilution, Millipore, Cat# MAB1568, RRID: AB_94259), anti-Calb1 (1:200 dilution, Cell Signaling Technology, Cat# 13176, RRID: AB_2687400), anti-Ctbp2 (1:500 dilution, BD Biosciences, Cat# 612044, RRID: AB_399431), anti-Shank1/2/3 (1:300 dilution, Santa Cruz Biotechnology, Cat# sc-393963), donkey anti-mouse Alexa Fluor 488 (1:500 dilution, Thermo Fisher Scientific, Cat# A-11059; RRID: AB_142495), goat anti-mouse IgG1 Alexa Fluor 488 (1:500 dilution, Thermo Fisher Scientific, Cat# A-21121, RRID: AB_2535764), goat anti-mouse IgG2a Alexa Fluor 647 (1:500 dilution, Thermo Fisher Scientific, Cat# A-21241, RRID: AB_2535810), donkey anti-rabbit Alexa Fluor 555 (1:500 dilution, Thermo Fisher Scientific, Cat# A-31572; RRID: AB_162543), donkey anti-goat Alexa Fluor 647 (1:500 dilution, Thermo Fisher Scientific, Cat# A-21447; RRID: AB_141844). Fluorescence-conjugated Phalloidin (1:500 dilution, Thermo Fisher Scientific, Cat# A22287) and DAPI (1:1000 dilution, Thermo Fisher Scientific, Cat# D1306) were also used.

### Cell counting

Z-stack images were acquired on a Leica TCS SP8 confocal microscope using either a 40× (numerical aperture 1.30) or 63× (numerical aperture 1.40) oil immersion objective and analyzed with Fiji software (v1.53, NIH, USA). For utricles, cells were manually counted in three distinct, non-overlapping 100 × 100 μm regions in both the striolar and extrastriolar zones (Fig. 1B). For cristae, cells were counted in three distinct, non-overlapping 50 μm × 50 μm regions in the central and peripheral zones (Fig. 3A). For saccules, cells were counted in three distinct, non-overlapping 50 μm × 50 μm regions in the striola and extrastriola zones (Figure S1A). Total HC numbers in different regions were calculated as the HC density (cells/µm^2^) multiplying the area (µm^2^).

We categorized the hair bundles of the surviving utricular HCs as long, short, and absent. Each type of hair bundle was counted separately in 100 μm × 100 μm regions in both the striola and extrastriola. For the quantification of nerve terminals in utricles, type I HCs or type II-like HCs (in the *Gfi1*^Cre^/*Atoh1*-OE group) with full or partial Tuj1^+^ calyxes were counted separately. A full calyx was defined when the nerve terminal encased at least 95% of the cell body circumference in the XY view or extended toward the apical neck. A partial calyx was defined as a nerve terminal that incompletely encased the HC (< 95%) in the XY view.

### Statistical analysis

Statistical analyses were performed using GraphPad Prism 8.0. Data are presented as mean ± SEM. Group comparisons were made as follows: two groups by unpaired two-tailed Student’s t-test; three or more groups by one-way ANOVA with Tukey’s post hoc test; non-parametric data (tail-hanging scores and swimming scores) by Mann-Whitney test. Simple linear regression was used for correlation analysis. A p-value < 0.05 was considered statistically significant.

## Supplementary Information

Below is the link to the electronic supplementary material.

Supplementary Material 1 (RAR 13.1 MB)

Supplementary Material 2 (DOCX 3.11 MB)

## Data Availability

The datasets generated and analyzed in the present study are available from the corresponding author on reasonable request. The manuscript has data included as electronic supplementary material.
