# Supplementary material for: Vestibular hair cell redundancy and the critical requirement of type I cells for balance maintenance
Source: Cell Mol Life Sci. 2026 Jul 17;83(1):376. doi: 10.1007/s00018-026-06348-1 (PMC13661832; doi:10.1007/s00018-026-06348-1)
Supplement: Supplementary file 1 [file 18_2026_6348_MOESM2_ESM.docx]

**Figure S1**

**Figure S1. DT injection induced dose-dependent injury of saccular HCs. Related to Figure 3.**

(A) Schematic of the saccule and its different zones, including the striola (S) and the extrastriola (ES). Squares indicate sampling regions for quantitative statistics.

(B) Low-magnification images showing a decrease in Myo7a^+^ HC density with increasing doses of DT.

(C) Representative images demonstrating a decrease in Myo7a^+^, Sox2^-^/Myo7a^+^, and Sox2^+^/Myo7a^+^ HCs in both the striola and extrastriola following increasing doses of DT

(D) Total HC counts expressed as percentages of the control group (n = 3, 4, or 5).

(E) The number of residual saccular HCs correlates with the doses of DT (simple linear regression, p < 0.0002).

(F-H) Counts of Myo7a^+^ HCs (F) (n = 3, 4, or 5), Sox2^-^/Myo7a^+^ type I HCs (G) (n = 3 or 4) and Sox2^+^/Myo7a^+^ type II HCs (H) (n = 3 or 4) in the striola and extrastriola expressed as percentages of the control group.

Scale bars: 100 µm in B; 10 µm in C. Red asterisks indicate statistical significance compared with the NS group. One-way ANOVA was performed for D, F-H. * p < 0.05, ** p < 0.01, *** p < 0.001, **** p < 0.0001.

**Figure S2**

**Figure S2 DT injection induced dose-dependent injury of vestibular HCs. Related to Figure 1, 3 and S1.**

(A) Myo7a+ HC counts in the striola and the extrastriola of the utricles expressed as percentages of the control NS group (n = 6 or 7).

(B) Myo7a+ HC counts in the striola and the extrastriola of the saccules expressed as percentages of the control NS group (n = 3, 4, or ­5).

(C) Myo7a+ HC counts in the central zone and the peripheral zone of the cristae expressed as percentages of the control NS group (n = 6 or 7).

(D) Sox2–/Myo7a+ type I HC and Sox2+/Myo7a+ type II HC counts in the striola of the utricles expressed as percentages of the control NS group (n = 3 or 4).

(E) Sox2–/Myo7a+ type I HC and Sox2+/Myo7a+ type II HC counts in the striola of the saccules expressed as percentages of the control NS group (n = 3 or 4).

(F) Sox2–/Myo7a+ type I HC and Sox2+/Myo7a+ type II HC counts in the central zone of the cristae expressed as percentages of the control NS group (n = 3 or 4).

(G) Sox2–/Myo7a+ type I HC and Sox2+/Myo7a+ type II HC counts in the extrastriola of the utricles expressed as percentages of the control NS group (n = 3 or 4).

(H) Sox2–/Myo7a+ type I HC and Sox2+/Myo7a+ type II HC counts in the extrastriola of the saccules expressed as percentages of the control NS group (n = 3 or 4).

(I) Sox2–/Myo7a+ type I HC and Sox2+/Myo7a+ type II HC counts in the peripheral zone of the cristae expressed as percentages of the control NS group (n = 3 or 4).

Red asterisks indicate statistical significance between the two groups marked by the straight lines. Data were analyzed by unpaired t-test. * p < 0.05, ** p < 0.01, *** p < 0.001, **** p < 0.0001.

**Figure S3**

**Figure S3. Synapses of the residual utricular HCs after DT treatment. Related to Figure 4.**

(A) Representative immunofluorescence images of Ctbp2, Shank1/2/3 and Myo7a.

(B) Quantification of Ctbp2^+^/ Shank1/2/3^+^paired synapses and orphan Ctbp2^+^ puncta (per HC). No significant differences were found among the three groups (n = 15 HCs from 3 mice).

Scale bars: 2 µm. One-way ANOVA was performed for B.

**Figure S4**

**Figure S4 HAVOR test results from DT-injected *Pou4f3*^DTR^ mice. Related to Figure 5.**

(A-E) Gain values in the HAVOR tests at various frequencies (n = 7, 8, 9, or 10).

Red asterisks indicate statistical significance compared with the NS group. One-way ANOVA was performed for A-E. * p < 0.05, ** p < 0.01, *** p < 0.001, **** p < 0.0001.

**Figure S5**

**Figure S5** **Quantification of Sox2⁻ type I and Sox2⁺ type II vestibular HCs in *Pou4f3*^DTR^ mice treated with 15 or 25 ng/g DT. Related to Figure 6.**

(A) The number of Sox2⁺ type II HCs was comparable between the two groups, whereas Sox2⁻ type I HCs were significantly reduced in the 25 ng/g DT group (n = 3 or 4).

Data were analyzed by unpaired t-test. ** p < 0.01; ns, not significant.

**Figure S6**

**Figure S6** **The effect of IDPN and *Atoh1* overexpression on stereociliary bundles and innervation of utricular HCs. Related to Figure 6.**

(A) High-magnification images of Phalloidin-labeled HC bundles. The white dashed lines mark HCs with short bundles.

(B) Statistical data showing the percentages of residual HCs with long, short, or no bundles (n = 3). Statistical significance for the percentages of HCs with long bundles is indicated on the graph.

Scale bars: 10 μm in A. Blue, grey, and orange asterisks indicate statistical significance compared with the respective controls. Black asterisks indicate statistical significance between the two groups marked by the straight lines. Unpaired t-test was performed in B. * p < 0.05, ** p < 0.01, *** p < 0.001, **** p < 0.0001, ns, not significant.

**Figure S7**

**Figure S7 IDPN injection or *Atoh1* overexpression reduced crista type I HCs. Related to Figure 6.**

(A-B) Low-magnification images showing Myo7a^+^ HC density in the cristae.

(C) Quantification of total crista HC numbers (n = 3, 6, or 7).

(D-E) Representative images demonstrating that most crista HCs in IDPN-treated mice (D) or *Gfi1*^Cre^/*Atoh1*-OE mice (E) were Sox2^+^ HCs in both the central and peripheral zones.

(F-H) Quantification of Myo7a^+^ HCs (F)(n = 3, 6 or 7), Sox2^–^/Myo7a^+^ HCs (G) (n = 3 or 4) and Sox2^+^/Myo7a^+^ HCs (H) (n = 3 or 4) in the central and peripheral zones.

Scale bars: 100 μm in A and B; 10 μm in D and E. Blue, grey, and orange asterisks indicate statistical significance compared with the respective controls. Black asterisks indicate statistical significance between the two groups marked by the straight lines. Unpaired t-test was performed for C, F-H. * p < 0.05, ** p < 0.01, *** p < 0.001, **** p < 0.0001, ns, not significant.

**Figure S8**

**Figure S8 IDPN injection or *Atoh1* overexpression reduced saccular type I HCs. Related to Figure 6.**

(A-B) Low-magnification images showing the density of Myo7a^+^ HCs in the saccules.

(C) Quantification of total saccular HC numbers (n = 3).

(D) Quantification of Myo7a^+^ HCs in the striola and extrastriola (n = 3).

(E-F) Representative images demonstrating that most saccular HCs in IDPN-treated mice (E) or *Gfi1*^Cre^/*Atoh1*-OE mice (F) were Sox2^+^ HCs in both the striola and extrastriola.

(G-H) Quantification of Sox2^–^/Myo7a^+^ HCs (G) and Sox2^+^/Myo7a^+^ HCs (H) in the striola and extrastriola (n = 3).

Scale bars: 100 μm in A and B; 10 μm in E and F. Blue, grey, and orange asterisks indicate statistical significance compared with the respective controls. Black asterisks indicate statistical significance between the two groups marked by the straight lines. Unpaired t-test was performed for C, D, G, H. *p < 0.05, **p < 0.01, *** p < 0.001, ****p < 0.0001, ns, not significant.

**Figure S9**

**Figure S9 HAVOR test results from 10ng/g DT-treated *Pou4f3*^DTR^, IDPN-injured and *Gfi1*^Cre^/*Atoh1*-OE mice. Related to Figure 8.**

(A-E) Gain values in the HAVOR tests at various frequencies (n = 6, 7, or 8).

Blue, grey, and orange asterisks indicate statistical significance compared with the respective controls. Black asterisks indicate statistical significance between the two groups marked by the straight lines. Unpaired t-test was performed for A-E. * p < 0.05, ** p < 0.01, *** p < 0.001, **** p < 0.0001.
